# A Premature Termination of Human Epidermal Growth Factor Receptor Transcription in *Escherichia coli*

**DOI:** 10.1155/2014/830923

**Published:** 2014-10-15

**Authors:** Jihene Elloumi-Mseddi, Karim Jellali, Antonio Villalobo, Sami Aifa

**Affiliations:** ^1^Centre of Biotechnology of Sfax, P.O. Box 1177, 3018 Sfax, Tunisia; ^2^Instituto de Investigaciones Biomédicas, Consejo Superior de Investigaciones Científica and Universidad Autónoma de Madrid, Arturo Duperier 4, 28029 Madrid, Spain

## Abstract

Our success in producing an active epidermal growth factor receptor (EGFR) tyrosine kinase in *Escherichia coli* encouraged us to express the full-length receptor in the same host. Despite its large size, we were successful at producing the full-length EGFR protein fused to glutathione S-transferase (GST) that was detected by Western blot analysis. Moreover, we obtained a majoritarian truncated GST-EGFR form detectable by gel electrophoresis and Western blot. This truncated protein was purified and confirmed by MALDI-TOF/TOF analysis to belong to the N-terminal extracellular region of the EGFR fused to GST. Northern blot analysis showed two transcripts suggesting the occurrence of a transcriptional arrest.

## 1. Introduction

Since its discovery, the epidermal growth factor receptor (EGFR) continues to be the subject of myriad investigations in cancer signalling. EGFR is a transmembrane protein composed of an extracellular region containing the ligand-binding site, a transmembrane *α*-helix, and an intracellular region harboring the tyrosine kinase domain, that is, flanked by a juxtamembrane domain (JM) and a C-terminal tail. The majority of solid tumor types overexpress at least one of the EGFR family members (HER1/EGFR, HER2/Neu, HER3, and HER4) [1]. As a consequence, the EGFR family has been targeted by many antibodies for therapeutic purpose, as many of them were approved to treat different cancer types (i.e., breast, colorectal, and non-small cells lung carcinoma (NSCLC), among others) [2]. Moreover, many tyrosine kinase inhibitors (TKI) competing with ATP binding were used to antagonize these receptors, and some of them were approved also for treating some cancer types, such as NSCLC [3]. Progress has been done in understanding the EGFR activation process especially with research aided by the crystallization of distinct segments of the receptor.

The ligand-induced dimerization of the EGFR was revealed by independent crystal structure studies showing that both juxtaposed monomers are maintaining a “butterfly”-like dimeric structure via a protruding loop from each domain II [4, 5].

Stamos et al. solved the EGFR tyrosine kinase crystal structure, encouraging the exploration of the relationship between the active ectodomain (ECD) conformational change and the intracellular activation of the tyrosine kinase (TK) domain [6]. Many studies have tried to understand the dimerization of the intracellular region of the EGFR leading to autophosphorylation showing the importance of the calmodulin-binding site located at the juxtamembrane (JM) segment in this process [7–9]. An allosteric mechanism of EGFR TK activation was proposed based on the formation of an asymmetric dimer of the EGFR kinase domain [10], where the JM region was shown to be the activator of the allosteric mechanism [11–13].

The ECD is subdivided into four domains; domains I and III are indispensable for ligand binding and domains II and IV play a crucial role in receptor dimerization and activation.

There are 12 potential N-linked glycosylation sites in the receptor, two in each of domains I and II and four in each of domains III and IV [14]. It was shown that the glycosylation process is related to ligand-binding acquisition [15], and core glycosylation plays an important role in both EGF binding and kinase activation [16]. Treatment with tunicamycin yielded a 130 kDa aglycosylated form of the EGFR with altered ligand-binding capacity, poor kinase activation, and aberrant subcellular localization of the receptor [17]. Interestingly, the combination of tunicamycin and erlotinib caused more inhibitory effect on EGFR phosphorylation than that induced by erlotinib alone [18]. Tsuda et al. suggested that the sugar chain linked to Asn-420 of the ECD plays a crucial role in EGF binding and prevents the spontaneous oligomerization of the EGFR [19], which otherwise may lead to uncontrollable receptor activation. Moreover, it was shown that core glycosylation is responsible for EGFRvIII self-dimerization [20].

Heterologous expression of the intracellular EGFR domain [21, 22] or the ECD of some EGFR family members was also attained in* Escherichia coli* and thereafter purified yielding an active recombinant tyrosine kinase [22] that allowed the preparation of antibodies [23, 24].

In the present work, we have modified the* E. coli* culture conditions in order to improve the quantity and quality of recombinant full-length and a truncated segment of the EGFR expressed as a fusion with the GST tag. The recombinant proteins could be used for the production of corresponding anti-EGFR antibodies.

## 2. Materials and Methods

### 2.1. Strains and Reagents

*E. coli* strain BL21 CodonPlus (Stratagene) was used for GST-fusion protein expression and JM109 competent bacteria (Promega) were used for plasmid construction and maintenance. The vector pLXSN, containing the full-length human EGFR, was a gift from Professor Axel Ullrich (Max Planck Institute, Martinsried, Germany).* E. coli* expression vector pGEX-6P-1 was purchased from Amersham Pharmacia Biotech. Anti-EGFR (sc-03) and anti-GST (sc-459) antibodies were obtained from Santa-Cruz Biotechnology. The horseradish peroxidase conjugated anti-rabbit and anti-mouse IgG antibodies were purchased from Promega.

### 2.2. Plasmid Construction

The DNA fragment coding for the full-length EGFR was amplified by PCR using Pfu-polymerase (Stratagene) and the pLXSN-EGFR plasmid as template. The following oligonucleotides were used for PCR amplification: 5′-GA GTC GAC CGA TGC GAC CCT CCG GGA C-3′, as forward primer, with a SalI site, underlined, and 5′-GA GCG GCC GCC CTC CGT GGT TCA TGC TCC A-3′, as a reverse primer, with a NotI site, underlined. The obtained fragment was double digested by SalI-NotI and inserted in pGEX-6P-1. We used S-300 columns (Amersham Pharmacia Biotech) to purify PCR products and a Qiagen kit (QIAquick PCR purification kit) to remove restriction enzymes from digested DNA before ligation using the “ready-to-go” T4 DNA ligase (Amersham Pharmacia Biotech). The resulting construct, named pGEX-EGFR, was analyzed by restriction enzymes and DNA sequencing to ascertain its correctness.

### 2.3. Recombinant Protein Expression Analysis and Dimerization

*E. coli* BL21 strains were grown overnight in Luria's broth or M9 minimal medium containing ampicillin (75 *μ*g/mL). IPTG induction, expression analysis, and GST proteins immobilization on glutathione-Sepharose 4B beads were performed as previously described [22].

The dimerisation assay was performed on 20 *μ*g of protein extract incubated with 150 *μ*M BS^3^ for 10 min at 4°C or 0.25 M glutaraldehyde for 1 min at room temperature. The reaction was stopped, respectively, by the SDS-PAGE loading buffer or 0.6 M glycine.

The proteins were then analysed on SDS-PAGE followed by a Western blot using anti-EGFR or anti-GST antibodies.

### 2.4. In-Gel Protein Digestion and Sample Preparation for MALDI-TOF/TOF Analysis

The bands of interest from Coomassie colloidal-stained 1D gel were excised manually, deposited in 96-well plates, and processed automatically in a Proteineer DP (BrukerDaltonics, Bremen, Germany). The digestion protocol used was based on Shevchenko et al. [25] with minor variations: gel plugs were washed first with 50 mM ammonium bicarbonate then with acetonitrile (ACN) prior to reduction with 10 mM dithiothreitol in 25 mM ammonium bicarbonate solution. Alkylation was carried out afterwards with 55 mM 3-*β*-indole acrylic acid (IAA) in 50 mM ammonium bicarbonate. Gel pieces were rinsed with 50 mM ammonium bicarbonate and thereafter with ACN and were dried under a stream of nitrogen. Modified porcine trypsin (sequencing grade; Promega, Madison, WI) was added at a final concentration of 16 ng/*μ*L in 25% ACN/50 mM ammonium bicarbonate solution and the digestion was incubated at 37°C for 6 h. The reaction was stopped by adding 0.5% trifluoroacetic acid (TFA). The tryptic-eluted peptides were dried by speed-vacuum centrifugation and were resuspended in 4 *μ*L of MALDI solution. A 0.8 *μ*L aliquot of each peptide mixture was deposited onto a 389-well OptiTOF Plate (Applied Biosystems, Framingham, MA) and allowed to dry at room temperature. Matrix solution (3 mg/mL *α*-cyano-4-hydroxycinnamic acid (CHCA) in MALDI solution) was then added (0.8 *μ*L) and the mixture was dried at room temperature.

### 2.5. MALDI Peptide Mass Fingerprinting, MS/MS Analysis, and Database Searching

For MALDI-TOF/TOF analysis, samples were automatically acquired in an ABi 4800 MALDI TOF/TOF mass spectrometer (Applied Biosystems, Framingham, MA) in positive ion reflector mode (the ion acceleration voltage was 25 kV for MS acquisition and 1 kV for MS/MS) and the obtained spectra were stored into the ABi 4000 Series Explorer Spot Set Manager. Peptide mass fingerprinting (PMF) and mass spectrometry/mass spectrometry (MS/MS) fragment ion spectra were smoothed and corrected to zero baseline using routines embedded in ABi 4000 Series Explorer Software v3.6. Each PMF spectrum was internally calibrated with the mass signals of trypsin autolysis ions to reach a typical mass measurement accuracy of <25 ppm. Known trypsin and keratin mass signals, as well as potential sodium and potassium adducts (+21 Da and +39 Da, resp.), were removed from the peak list. To submit the combined PMF and MS/MS data to MASCOT software v.2.1 (Matrix Science, London, UK), GPS Explorer v4.9 was used, searching in the nonredundant NCBI protein database.

### 2.6. *E. coli* Total RNA Extraction and Northern Blot Analysis

After solubilization, cells were solubilized in Eurosol (EuroClone) solution and chloroform was added in the proportion of 1 : 10. After mixing and 5-minute incubation on wet ice, the sample was centrifuged for 15 min at 12,000 g. The upper aqueous phase containing RNA was then collected and precipitated with cold isopropanol for 15 min on wet ice. After centrifugation at 12,000 g at 4°C, the total RNA pellet was washed with 75% ethanol and centrifuged at 8,000 g for 15 min at 4°C. Northern blot analysis was performed according to Sambrook and Russel [26]. A PCR fragment corresponding to the EGFR ECD was [^32^P]dCTP-radiolabelled by Rediprime II Random Prime Labelling System (Amersham) and used as a probe to detect EGFR messengers.

## 3. Results

### 3.1. Expression of Recombinant EGFR in Fusion with GST

**Figure 1:**
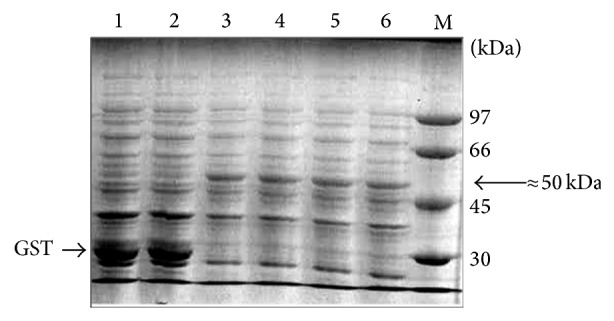
GST-EGFR expression in* E. coli* BL21 strain. The proteins were separated in a 10% SDS-PAGE and stained with Coomassie blue. (Lanes 1 and 2) Two clones of* E. coli* BL21 cells transformed by empty pGEX-6P-1 vector. (Lanes 3–6) Different clones of* E. coli* BL21 cells transformed by pGEX-EGFR. (Lane M) The protein masses of the BioRad markers are indicated in kDa. The arrows point to GST and the ≈50 kDa protein.

**Figure 2:**
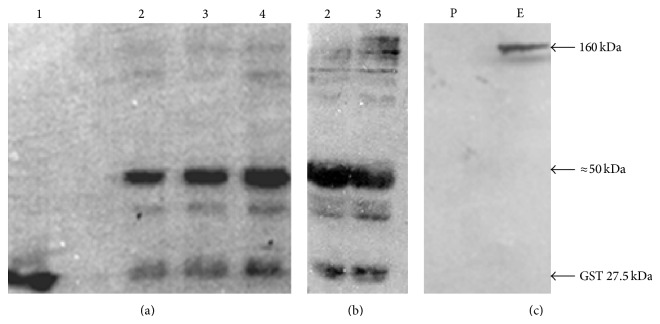
Western blot analysis of GST-EGFR. Samples from* E. coli* expressing GST (lane 1) and GST-EGFR (lanes 2–4) were processed by Western blot with the anti-GST antibody. (a) 5 min ECL exposure; (b) 30 min ECL exposure; (c) the protein extract was immobilized on glutathione-Sepharose beads, run on SDS-PAGE, and analyzed by Western blot using the anti-EGFR antibody. (Lane P) Protein extract of* E. coli* cells transformed with empty pGEX-6P-1. (Lane E) Protein extract of* E. coli* cells transformed with pGEX-EGFR after immobilization on glutathione beads. Arrows indicate the position of each expressed recombinant protein.

The DNA fragment encoding the open reading frame (ORF) of the EGFR was amplified using pLXSN-EGFR as template and inserted in frame with the ORF of the GST in pGEX-6P-1. The obtained construct, pGEX-EGFR, was used to transform BL21 CodonPlus. In order to optimize the expression of full-length GST-EGFR, we have varied the culture media and analyzed the produced proteins in each condition by Western blot. After culture/induction of the recombinant strains as described in Section 2, cells were solubilized in loading buffer and total proteins were analyzed in SDS-PAGE. The analysis displays a major ≈50 kDa band corresponding to a truncated form of the EGFR (Figure 1). The detection of this truncated form was confirmed by Western blot analysis using the anti-GST as primary antibody (Figure 2(a)). Moreover, a longer exposition to ECL allowed the detection of a 160 kDa band corresponding to the full-length GST-EGFR (Figure 2(b)). The full-length GST-EGFR was also detected by the anti-EGFR antibody, especially after immobilization in glutathione beads of the recombinant protein derived from* E. coli* grown in M9 minimal medium (Figure 2(c)).

The dimerization of the receptor was also studied via BS^3^ chemical cross-linking and glutaraldehyde. Our results showed that the full-length GST-EGFR preserved the ability of forming dimers even in the absence of EGF (data not shown).

### 3.2. Analysis of the GST-EGFR Truncated Form

**Figure 3:**
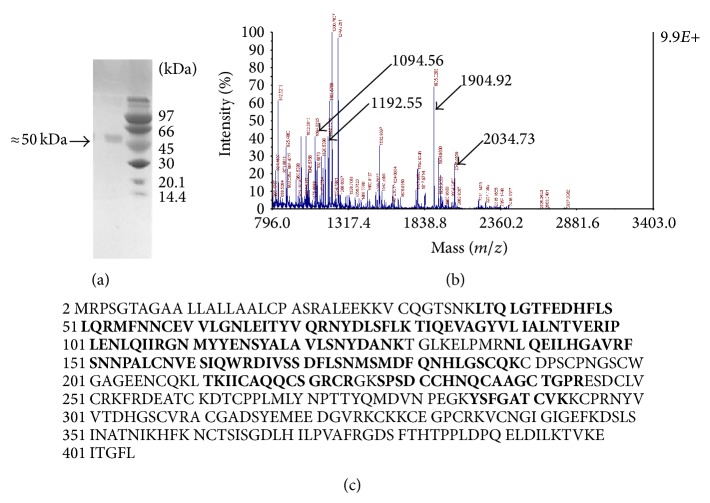
Identification of the truncated protein by MALDI-TOF/TOF analysis. (a) The gel in the inset shows the purified N-terminal truncated EGFR with its corresponding size. The protein masses of the BioRad markers are indicated in kDa. (b) The spectrum shows different peaks of the MS analysis done by MALDI-TOF. The N-terminal truncated EGFR (≈50 kDa) was subjected to in-gel trypsin digestion before MALDI-TOF analysis. The arrows show the peaks that served for MS/MS analysis with their corresponding masses used for protein identification. (c) MASCOT results: matched peptides to EGFR sequence are shown in capital bold; the last identified peptide ends at K293.

In order to understand the origin of EGFR truncation and check the integrity of its ORF, the ≈50 kDa truncated GST-EGFR protein overproduced in all media used was purified from the SDS-PAGE as described in Section 2. After trypsin digestion, this protein was subjected to MALDI-TOF/TOF analysis (Figure 3). Many peaks from the obtained spectrum were identified as peptides belonging to the GST or the EGFR proteins using the Mascot search tool (http://www.matrixscience.com/) (Figure 3). The identification of the obtained peptides showed that the truncated protein starts from the GST and stops at domain II of the EGFR according to the matched peptides (Figure 3(c)). According to the last matched peptide, the truncation corresponds to the first 293 amino acids of EGFR protein sequence which becomes 530 residues with the GST. The size of this truncated protein (≈50 kDa) was determined by the Expazy proteomic tools (PeptideMass at http://www.expasy.org/tools/). The corresponding mRNA size was 1590 bp (879 bp corresponds to the EGFR RNA).

**Figure 4:**
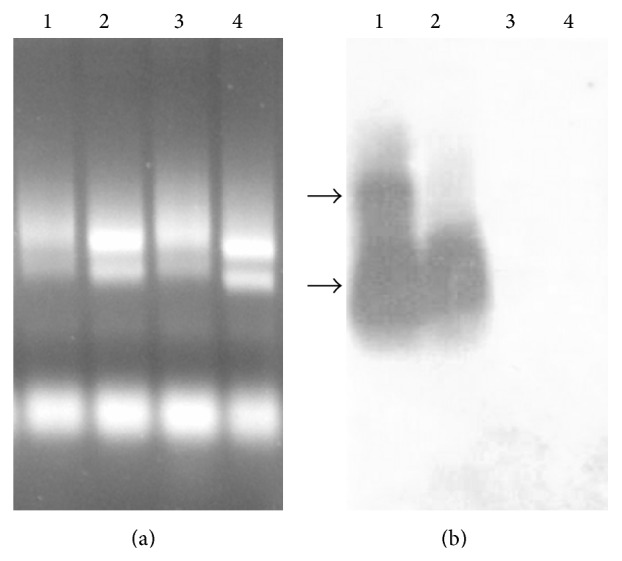
Northern blot analysis of EGFR expression. (a) Total RNA (20 *μ*g) was loaded on a formaldehyde 1.5% agarose gel. RNA was extracted from* E. coli* BL21 cells transformed with pGEX-EGFR after one-hour induction with 0.1 mM IPTG (lane 1) or without induction (lane 2). Extracted RNA of* E. coli* BL21 cells transformed with empty pGEX-6P-1 after one-hour induction with 0.1 mM IPTG (lane 3) or without IPTG induction (lane 4) used as control. (b) The gel in A was blotted on a nitrocellulose membrane and radioactive hybridization was performed with a 1.9 kb PCR fragment of EGFR ECD cDNA.

Northern blot analysis using a PCR probe corresponding to the EGFR ECD showed the presence of two transcripts. The small one is present even in noninduced cells but the large one is produced after IPTG stimulation only (Figure 4).

These results suggest that the largest transcript encodes the full-length GST-EGFR while the smaller one, expressed even in the absence of IPTG induction, is probably encoding the truncated protein. The presence of the small transcript under noninduced condition could be explained by the “leaky” expression encountered with the pGEX vectors as described by the supplier.

## 4. Discussion

The production of EGFR protein in an easy and less costing host could facilitate enormously the production of specific antibodies or screening for tyrosine kinase antagonists. For long time* E. coli *has been an ideal heterologous expression host characterized by the multiplicity of developed strains and the continuous production/purification improvement of the recombinant proteins. We have previously succeeded with the expression and production of an active intracellular EGFR domain [22], which encouraged our present work. We now achieved expressing the full-length EGFR as a GST-fusion protein that was detectable only by Western blot analysis. Moreover chemical cross-linking with BS^3^ showed that EGFR dimerization is preserved in* E. coli*.

The expression of EGFR was concomitant with the production of the ≈50 kDa truncated protein that was analyzed in MALDI-TOF/TOF, and the MASCOT search confirmed its identity corresponding to a truncation within the ECD domain II of the receptor. The origin of this truncation was analyzed by Northern blot, and the presence of two transcripts was detected: a larger one that was dependent on IPTG stimulation of the promoter and is more probably encoding the full-length GST-EGFR protein and a shorter transcript that is present even in the absence of IPTG induction and could encode the truncated protein. The presence of this small transcript in noninduced cells is very likely due to a premature transcriptional termination signal within the EGFR cDNA, although this arrest was not total since the detection of the full-length transcript and protein was apparent. This could be explained by the existence of a transcription attenuation phenomenon due to* E. coli*-like leaky terminator signals within the EGFR cDNA sequence. We have scanned the EGFR cDNA sequence by several online programs and found two overlapping hairpin structures resembling those of the tryptophan operon. In fact, the sequence of EGFR cDNA (starting from the ATG) 1220–ttcaggct-1227 hybridizes with the sequence 1331-aagtccga-1324 in a first palindrome and with the sequence 1236-aagtccg-1230 in another palindrome. Favoring one of these two palindromes could be responsible for the present transcription attenuation.

Further work should be performed to establish the molecular basis of this premature termination in transcription of the full-length EGFR and on the possibility of bypassing this arrest in order to express larger quantities of the full-length receptor. Meanwhile, the overexpression of the 50 kDa truncated protein could serve itself for the production of anti-EGFR antibodies directed against the N-terminal of the receptor.

## 5. Conclusions

The adoption of* E. coli* as a host of heterologous expression is still a primordial strategy for any gene functional or immunogenicity study. The present work demonstrates that this strategy is valuable also for transmembrane proteins like receptor tyrosine kinases, which could facilitate proteomic studies. Moreover, encountering an* E. coli*-like transcription arrest signal within a human oncogene could have many impacts. Since EGFR has its viral homolog, ErbB, one can speculate the occurrence of DNA transfer from prokaryotic to eukaryotic genomes through viruses. We have to search for analogous results of expression to check our hypothesis.
